# One-Week High-Intensity Interval Training Increases Hippocampal Plasticity and Mitochondrial Content without Changes in Redox State

**DOI:** 10.3390/antiox9050445

**Published:** 2020-05-21

**Authors:** Jonathas Rodrigo dos Santos, Mariza Bortolanza, Gustavo Duarte Ferrari, Guilherme Pauperio Lanfredi, Glauce Crivelaro do Nascimento, Ana Elisa Calereiro Seixas Azzolini, Elaine Del Bel, Alline Cristina de Campos, Vitor Marcel Faça, Anderson Vulczak, Luciane Carla Alberici

**Affiliations:** 1 Departamento de Ciências BioMoleculares, Faculdade de Ciências Farmacêuticas de Ribeirão Preto, Universidade de São Paulo, Ribeirão Preto, São Paulo 14040-903, Brazil; jonathas.rodrigo.santos@usp.br (J.R.d.S.); gustavoduartef@usp.br (G.D.F.); anael@usp.br (A.E.C.S.A); 2 Departamento de Biologia Básica e Oral, Faculdade de Odontologia de Ribeirão Preto, Universidade de São Paulo, Ribeirão Preto, São Paulo 14040940, Brazil; marizabortolanza@yahoo.com.br (M.B.); glauce.nascimento@usp.br (G.C.d.N.); eadelbel@usp.br (E.D.B.); 3 Departamento de Bioquímica e Imunologia, Faculdade de Medicina de Ribeirão Preto, Universidade de São Paulo, Ribeirão Preto, São Paulo 14049-900, Brazil; lanfredi@usp.br (G.P.L.); vmfaca@gmail.com (V.M.F.); 4 Departamento de Farmacologia, Faculdade de Medicina de Ribeirão Preto, Universidade de São Paulo, Ribeirão Preto, São Paulo 14049-900, Brazil; allinecampos@usp.br

**Keywords:** ROS, exercise, oxidative stress, neurogenesis, brain

## Abstract

Evidence suggests that physical exercise has effects on neuronal plasticity as well as overall brain health. This effect has been linked to exercise capacity in modulating the antioxidant status, when the oxidative stress is usually linked to the neuronal damage. Although high-intensity interval training (HIIT) is the training-trend worldwide, its effect on brain function is still unclear. Thus, we aimed to assess the neuroplasticity, mitochondrial, and redox status after one-week HIIT training. Male (C57Bl/6) mice were assigned to non-trained or HIIT groups. The HIIT protocol consisted of three days with short bouts at 130% of maximum speed (Vmax), intercalated with moderate-intensity continuous exercise sessions of 30 min at 60% Vmax. The mass spectrometry analyses showed that one-week of HIIT increased minichromosome maintenance complex component 2 (MCM2), brain derived neutrophic factor (BDNF), doublecortin (DCX) and voltage-dependent anion-selective channel protein 2 (VDAC), and decreased mitochondrial superoxide dismutase 2 (SOD 2) in the hippocampus. In addition, one-week of HIIT promoted no changes in H_2_O_2_ production and carbonylated protein concentration in the hippocampus as well as in superoxide anion production in the dentate gyrus. In conclusion, our one-week HIIT protocol increased neuroplasticity and mitochondrial content regardless of changes in redox status, adding new insights into the neuronal modulation induced by new training models.

## 1. Introduction

Evidence has shown that neurological and mental illnesses may be delayed or avoided by regular exercise, being also useful in the treatment of neuronal diseases [1,2,3]. In this regard, exercise reduces the risk of dementia, stress, depression, and enhances cognitive functions and metabolic control [1]. The effects of exercise on neural plasticity have been observed through changes in neurochemistry and electrophysiological activity, increased neurogenesis, and the number and length of dendritic dendrites and spines [4,5].

Although exercise can have global effects on the brain, the region most affected by exercise is the hippocampus [1,6]. The hippocampus is a critical brain structure for learning and memory [7,8,9] and experimental models have shown that exercise is responsible for an increase in the volume and blood flow in the hippocampus [10]. Accumulation of evidence has shown that exercise influences neurogenesis in the dentate gyrus in the hippocampus [4,11], increases synapse plasticity, and promotes morphological changes in dendrites [11]. The neurogenesis in the hippocampus occurs through neuronal stem-cell proliferation in the subgranular zone of the dentate gyrus, differentiating, and maturating into granule cells before becoming incorporated into hippocampal neuronal networks [12]. Many intrinsic and extrinsic factors that can regulate neurogenesis and oxidative status are considered important regulators of neuronal plasticity [12,13].

Nevertheless, oxidative stress is usually linked to neuronal damage with synaptic losses, reduced neurotransmitters, and neuronal cell death [13,14]. However, physical exercise capacity to modulate the neuronal plasticity is linked with redox status [13,14,15]. Physical exercise reduces H_2_O_2_ generation and lipid peroxidation in the hippocampus, and enhances the neuronal number and planimetric volumes of the CA1 pyramidal layer [16,17]. Thus, exercise improves the brain’s antioxidant capacity, especially in the hippocampus. Interestingly, the antioxidant effect of exercise seems to be dependent on brain region [18]. While in some brain regions physical exercise may increase reactive oxygen species (ROS )levels, no relationship was established with oxidative damage [19].

Although many studies have demonstrated the neuroprotective effects of moderate aerobic exercise [20,21,22], the effects of high-intensity interval training (HIIT), marked by alternating short-terms of very intense anaerobic exercise with recovery periods [23], are still scarce. While in some studies with animal models, HIIT training over eight weeks showed no effect on neuroplasticity [6], others have shown HIIT training-promoted hippocampal neurogenesis [24], enhanced brain-derived neurotrophic factor (BDNF) action [25], and increased antioxidant capacity in the brain [26,27]. Although HIIT performed greatly in physical training programs [28], its effect is not yet fully understood on brain modulation. In this regard, our objective was to assess the neuroplasticity, mitochondrial content, and redox status after high-intensity interval training.

## 2. Materials and Methods

### 2.1. Animals

Male C57BL-6 mice (3 months old 22 ± 1.0 g) purchased from the vivarium of the University of São Paulo, Campus of Ribeirão Preto) were housed four per cage with free access to food and water and maintained on a 12 h light/12 h dark cycle at 22 ± 2 °C without humidity control. The animals were divided into four groups: control (sedentary one week), HIIT (one week training), control (sedentary five week), and HIIT (five week training). Control and HIIT groups were exposed to the same environmental conditions. All procedures were approved by the Ethics Committee on Use of Animals (CEUA no. 16.1.347.60.1).

### 2.2. Exercise Training Program

**Figure 1:**
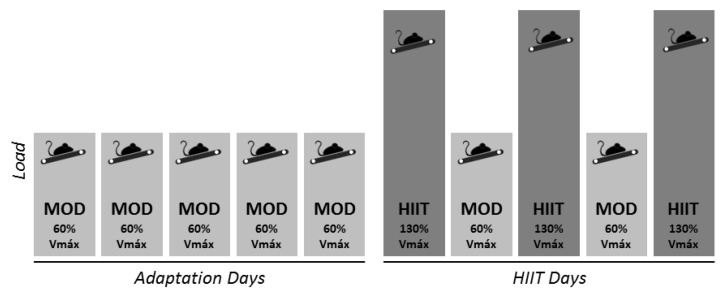
Experimental design. After one week adaptation in continue moderate intensity, one group exercised one week with high-intensity interval training (HIIT), while the other group exercised five weeks with HIIT. HIIT intensity was around 130% of the maximum speed and moderate intensity was around 60% of the maximum speed achieved in the incremental load test.

All exercise programs were performed in the animal’s dark period. Mice were first submitted to an incremental load test with warm-up for 15 min at a speed of 12 m min^−1^. The test itself began at a speed of 9 m min^−1^ and the speed was increased by 2 m min^−1^ every two minutes [29,30]. In the training protocol, the animals ran one week between 15−45 min continuously at 60% of the maximum speed (mean speed 14 m/min) reached in the incremental test load. Each HIIT session consisted of four to eight 30 s bouts at 130% of maximum speed achieved in the incremental load test (mean speed 30 m/min), followed by 2 min active rest phases at 40% of the maximum speed achieved in the test incremental load (mean speed 9 m/min). Three HIIT sessions were performed intercalated with moderate intensity continuous exercise sessions. The moderate intensity session consisted of 30 min at 60% of the maximum speed achieved in the incremental load test (Figure 1).

### 2.3. Western Blotting Analysis

Hippocampi were homogenized (20 mg/0.4 mL) in 0.1 M Tris-HCl (pH 7.4) containing protease and phosphatase inhibitors. The protein concentration was determined by the Bradford method [31] and then mixed with sodium dodecyl sulfate and polyacrylamide gel (SDS-PAGE) sample buffer. An equal amount of proteins was separated by SDS-PAGE and transferred to polyvinylidene difluoride membranes. Proteins were detected using antibodies against phospho-Akt (Ser 437)) and BDNF. Akt was from Cell Signaling Technology (Boston, MA, USA) and BDNF from Santa Cruz Biotechnology Inc. The results were normalized using β-actin (Cell Signaling Technology, Danvers, MA, USA) and presented as arbitrary units (A.U.) A similar procedure was used in previous studies [32,33].

### 2.4. Hydrogen Peroxide Generation

The hippocampus was homogenated in 0.1 M phosphate buffer (pH 7.4) and ~0.25 mg/mL was added to 0.2 mL of the same buffer containing 10 mM glutamate, 10 mM pyruvate, 4 mM malate, and 1 U/mL peroxidase at 37 °C. The reaction trigger was initiated by the addition of 2 µM Amplex Red, and hydrogen peroxide (H_2_O_2_) production was monitored by spectrofluorimetry at 563/587 nm (ex/em) [34] in a Model Synergy 2 fluorescence spectrophotometer (Biotek, Winooski, VT, USA) with continuous stirring. The results were analyzed through the slope variation rate, calculated by the difference between final fluorescence and initial fluorescence (unit of fluorescence—UF) divided by time and normalized by protein concentration analyzed by the Bradford protocol [31].

### 2.5. Protein Carbonylation Assay

Protein carbonyl content was determined by the method by Colombo et al. [35]. The assessment of carbonyl formation was conducted on the basis of the formation of protein hydrazone by reaction with 2,4-dinitrophenylhydrazine (DNPH). The hippocampus was homogenized in 0.1 M Tris-HCl buffer (pH 7.4). Homogenates were then treated with 10 mM DNPH (in HCl 2.0 M) for 1 h at room temperature; 10% trichloracetic acid was added, and the samples were centrifuged at 5000× *g* at 4 °C for 5 min. The pellet was washed with 20% trichloracetic acid, then three times with ethanol:ethyl acetate (1:1), dissolved with 6 M guanidine hydrochloride, and incubated for 30 min at 37 °C. The absorbance was measured at 366 nm. The protein carbonyl content was expressed as nmol carbonyl/mg protein using the molar absorption coefficient of DNPH (22,000 M^−1^ cm^−1^). The total protein concentration was obtained by the bicinchoninic acid protein assay method [36].

### 2.6. Relative Protein Quantification by Liquid Chromatography Coupled with Tandem Mass Spectrometry (LC-MS/MS)

For the sample preparation for relative protein quantification by LC-MS/MS, the hippocampus biopsies (homogenized in RIPPA buffer) were first denatured with 8 M urea in 100 µM Tris-HCl buffer (pH 8.5), reduced with 0.1 M DTT, alkylated using 0.5 M iodoacetamide, and digested by 40 µg of trypsin [37,38]. Each sample was injected in triplicate through the Xevo TQS (Waters) liquid chromatographic separation-tandem mass spectrometry (LC-MS/MS) system. Chromatographic separation was carried out by ultraperformance liquid chromatography (UPLC I-Class, Waters, Milford, MA, USA) using a C_18_ column (1.8 µm piece size, 100 Å pore size, 1 × 150 mm, Waters, Milford, MA, USA) in a linear gradient of 5–30% acetonitrile (in water and 0.1% formic acid) over 30 min at 100 µL/min. Detection of proteotypic peptides was performed through 3–5 fragments/transitions per peptide during a 2 min time window. The proteins analyzed were synapsin-1 (Syn1); sodium-dependent glutamate/aspartate transporter 1 (GLAST); proliferation marker protein Ki67 (Ki67); microtubule-associated protein 2 (MAP2); minichromosome maintenance complex componente 2 (MCM2); neuronal nuclei (NeuN); nestin (Nestin); doublecortin (DCX); brain derived neutrophic factor (BDNF); Hu-antigen R (HuR); superoxide dismutase 2, mitochondrial (SOD 2); and voltage-dependent anion-selective channel protein 2 (VDAC). The analysis was performed using the Skyline 3.5 program [39]; see Supplementary Table S1 for a list of proteins/peptides.

### 2.7. Immunohistochemistry Assay and Imaging

After one and five weeks, the mice were anesthetized with 10% ketamine (80 mg/kg) and 4% xylazine (10 mg/kg) and perfused with 4% paraformaldehyde. Brains were removed and post-fixed in 4% paraformaldehyde solution for 24 h and cryoprotected in a 30% sucrose solution 0.1 M phosphate buffer during 30 h. Brains were then frozen in isopentane (−40 °C, Sigma-Aldrich, St. Louis, MO, USA) and stored at −80 °C until histological processing. Serial coronal sections (30 µm) were cut using a cryostat (Cryocut, 1800, Leica, Heerbrugg-Switzerland) throughout the rostrocaudal extent of the hippocampus. The quantification of doublecortin (DCX) positive cells was conducted from a 1-in-6 series of hippocampal sections with 8–10 hippocampal sections spaced 180 µm apart, and corresponding to the hippocampal extension according to the following coronal coordinates from the bregma: −0.94 to −2.7mm [40]. For DCX immunohistochemistry, free floating sections were incubated in citrate buffer (60 °C, 30 min) and washed with Phosphate-Buffered Saline (PBS) + 0.15% Triton × 100. Endogenous peroxidases were inhibited with 1% H_2_O_2_ incubation for 30 min followed by 2% bovine serum albumin (BSA) and 5% goat serum for 60 min to block non-specific reactions.

Sections were incubated overnight with primary antibodies (rabbit anti-doublecortin 1:6000, sc-271390, Santa Cruz Biotechnology, Dallas, TX, USA), followed by 90 min of incubation of biotinylated secondary antibody (goat anti-rabbit; 1:1000, A6154, Vector Laboratories, Burlingame, CA, USA). Sections were processed by the avidin–biotin–peroxidase complex for 2 h (Vectastain ABC kit, Vector Laboratories, Burlingame, CA, USA.) and the immunoreactivity was revealed by the addition of diaminobenzidine (Sigma-Aldrich, San Luis, MO, USA) as the chromogen. The slices were mounted on slides and cover slipped for microscopic observations.

DCX^+^ cells were analyzed by light microscopy (Leica, 40×), in which the total number of DCX^+^ cells present in the SGZ of the dentate gyrus was measured. DCX^+^ cells were quantified across the entire granule cell layer and subgranular zone (~20 µm wide) from two dorsal sections (2 hemispheres). The granule cell layer volume was calculated by multiplying the section thickness (30 µm) by the 2D area (measured images with ImageJ softwre, version 1.8.0_112, Research Service Branch, National Institute of Mental Health, Bethesda, MD, USA), which was then used to calculate the DCX^+^ cell densities. 

### 2.8. Superoxide Anion Detection in Dentate Gyrus

For superoxide anion measurement, an additional group of mice was deeply anesthetized and decapitated. The brain was quickly removed and embedded in Tissue-tek^®^ (Alphen aan den Rijn, NL, USA) and immediately frozen at −40 °C. Coronal sections (16 µm) of the hippocampus were cut using a freezing microtome (LeicaR, model CM1850). The quantification was conducted from a 1-in-6 series of hippocampal sections with –810 hippocampal sections spaced 180 µm apart and corresponding to the hippocampal extension according to the following coronal coordinates from the bregma: −0.94 to −2.7 mm. Sections were incubated with dihidroetideo (DHE, 10 mM) for 15 min in a dark and humid chamber. Then, the sections were washed with saline solutions and fixed in 4% paraformaldehyde. DHE reacted with superoxide anion (O^2−^.) present in the tissues, producing 2-hidroxietideo and ethidium with a red fluorescence emission. Using a fluorescence microscope (Leica Imaging System Ltd., Cambridge, UK) the images of the sections were photographed (40×) and ImageJ software (version 1.8.0_112, Research Service Branch, National Institute of Mental Health, Bethesda, MD, USA) was used to quantify the intensity of the emitted red fluorescence.

### 2.9. Data Analysis

All statistical analyses were performed using GraphPad Prism™5.0 (GraphPad, La Jolla, CA, USA). Variables were compared using Analysis of variance (ANOVA) and Bonferroni post-hoc test between four groups and the two-tailed unpaired Student’s *t*-test between two groups. The data were presented as mean ± standard error of the mean (SEM), and *p*-value < 0.05 was considered as significant.

## 3. Results

### 3.1. High intensity Interval Training (HIIT) Modulated Hippocampal Neuroplasticity and Mitochondrial Content

**Figure 2:**
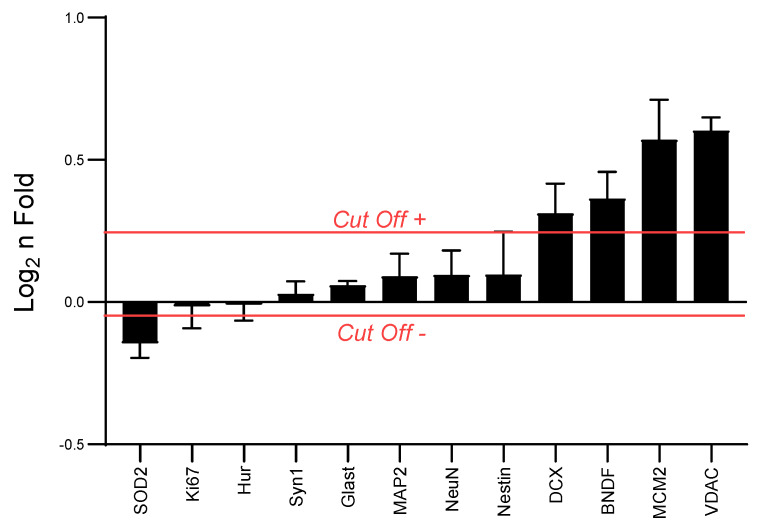
Effects of one-week HIIT on neuronal plasticity in the hippocampus. Proliferative and growth stem cell related proteins: Ki67, minichromosome maintenance complex componente 2 (MCM2), Nestin, Hu-antigen R (HuR), and sodium-dependent glutamate/aspartate transporter 1 (GLAST). Immature neurons and neurogenesis related proteins: doublecortin (DCX). Mature neuron related protein: neuronal nuclei (NeuN); Neuritogenesis related protein: microtubule-associated protein 2 (MAP2). Survival and neurogenesis: brain derived neutrophic factor (BDNF). Synaptogenesis related protein: synapsin-1 (Syn1). Mitochondrial transporter and mitochondria content marker: voltage-dependent anion-selective channel protein 2 (VDAC). Mitochondrial antioxidant enzyme: superoxide dismutase 2, mitochondrial (SOD 2). Cut-off line indicates the log2 fold-change threshold considering the mean of proteins expressed in the trained group in relation to the control group. Values obtained as ion peak area/α-tubulin peak area. Cut Off +: mean of the protein higher peak area in liquid chromatography coupled with tandem mass spectrometry (LC-MS/MS). Cut Off −: mean of the protein lower peak area in LC-MS/MS. Control *n* = 3, Trained *n* = 3.

High intensity interval training over one week increased the content of protein linked neuroplasticity in the hippocampus. The mass spectrometry analyses showed increased protein markers of cell proliferation (MCM2) [41], immature neuron content (DCX) [42], neuronal survival/neurogenesis (BDNF) [1], and mitochondrial content (VDAC) [43] in a trained group when compared to the non-trained condition (control) (Figure 2). No changes were observed in the content of Ki67, Nestin, HuR, Glast, Syn1, NeuN, and MAP2. Furthermore, the mitochondrial superoxide dismutase (SOD2), responsible for the dismutation of superoxide radical anion for hydrogen peroxide [44], was reduced in the hippocampus of trained animals.

**Figure 3:**
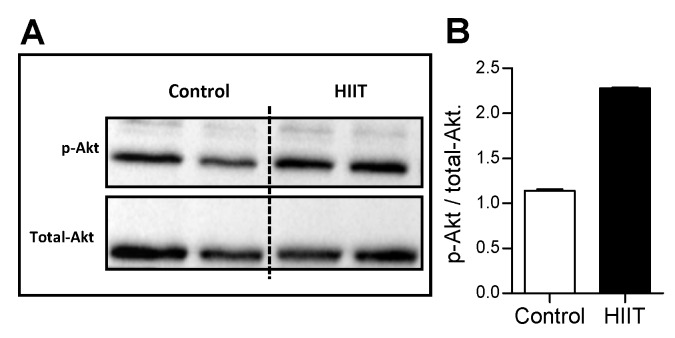
Effect of one-week HIIT on Akt phosphorylation in the hippocampus. (**A**) Immunoblotting and (**B**) blot densitometry ratio of phosphorylated (p-Akt) and total-Akt in hippocampus (control *n* = 2; HIIT *n* = 2) of the control and trained animals. Statistical analysis were performed by the Student’s *t*-test using *p* < 0.05.

In addition, after one week of HIIT, the ratio of phospho-Akt to total-Akt showed an upward trend in the hippocampus of the trained group (Figure 3A,B).

### 3.2. One-Week HIIT Did Not Modulate Redox Status in the Hippocampus and Dentate Gyrus

**Figure 4:**
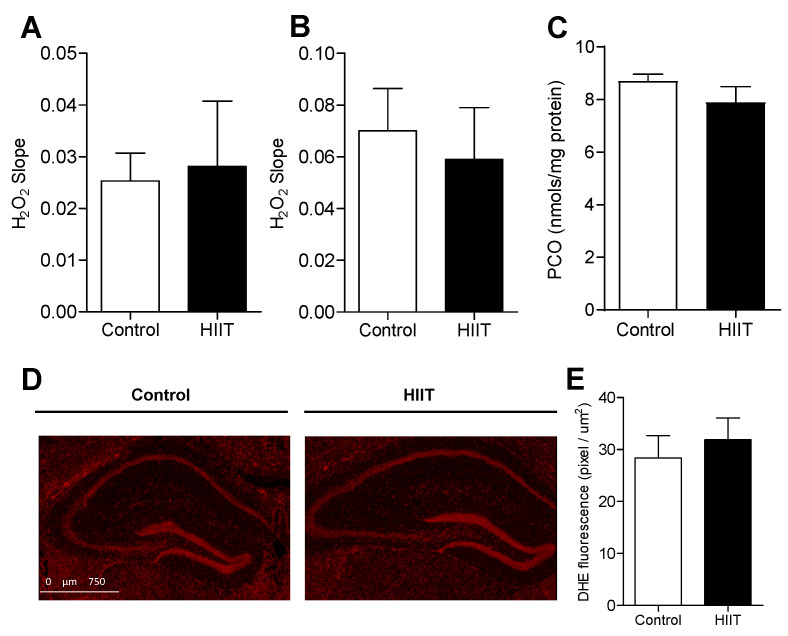
Effect of one-week HIIT on redox parameters in the hippocampus and dentate gyrus. (**A**) H_2_O_2_ production in the hippocampus and (**B**) cerebral cortex (control *n* = 3; HIIT *n* = 3); (**C**) carbonylated protein concentration in the hippocampus (control *n* = 4; HIIT *n* = 4); (**D**) DHE fluorescence—superoxide anion; (**E**) superoxide anion level in the dentate gyrus (control *n* = 4; HIIT *n* = 4); Slope: variation rate of UF/time, normalized by protein content (UF: fluorescence units). Statistical analyses were performed by the Student’s *t*-test using *p* < 0.05.

The promotion of neuroplasticity after one week of high-intensity interval training occurred regardless of changes in the redox state. The analyses showed no differences in the production of hydrogen peroxide (H_2_O_2_) in the hippocampus (Figure 4A) and cortex (as a comparative neural tissue) (Figure 4B), neither in the changes in a marker of oxidative stress in the hippocampus (carbonyl protein content, Figure 4C) or DHE-oxidation levels (superoxide anion, SO) in the dentate gyrus (Figure 4D,E) between the control and trained animals.

### 3.3. HIIT along Five Weeks Reduced Superoxide Anion and Did Not Modulate DCX^+^ Cells in Dentate Gyrus

**Figure 5:**
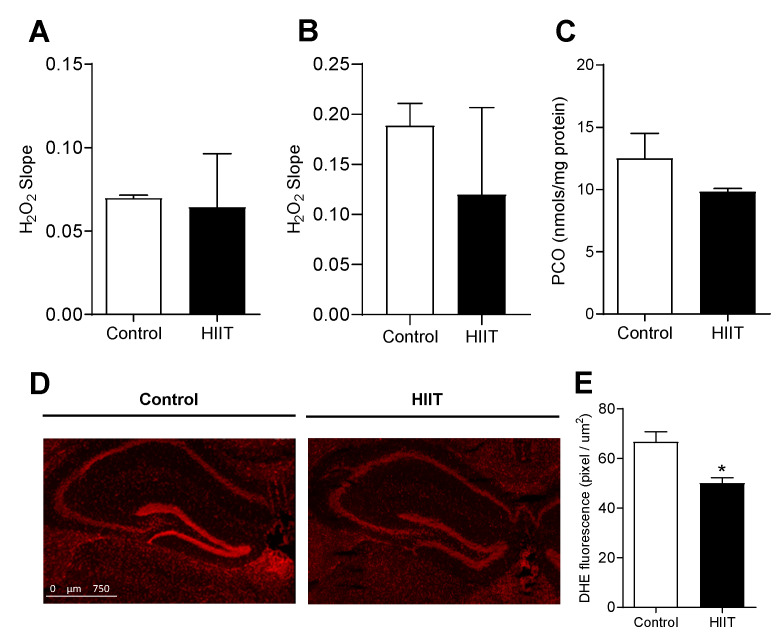
Effect of five-week HIIT on the redox state in the hippocampus and dentate gyrus. The same HIIT protocol training performed during the one-week analysis was repeated over five weeks. (**A**) hydrogen peroxide (H_2_O_2_) production in the hippocampus and (**B**) cerebral cortex (control *n* = 3; HIIT *n* = 3); (**C**) carbonylated protein concentration in the hippocampus (control *n* = 3; HIIT *n* = 2); (**D**) dihidroetideo (DHE) fluorescence—superoxide anion; (**E**) superoxide anion production in dentate gyrus (control *n* = 4; HIIT *n* = 4); Slope: variation rate of UF/t normalized by protein content (UF: unit of fluorescence). Statistical analyses were performed by the Student’s *t*-test using * *p* < 0.05.

**Figure 6:**
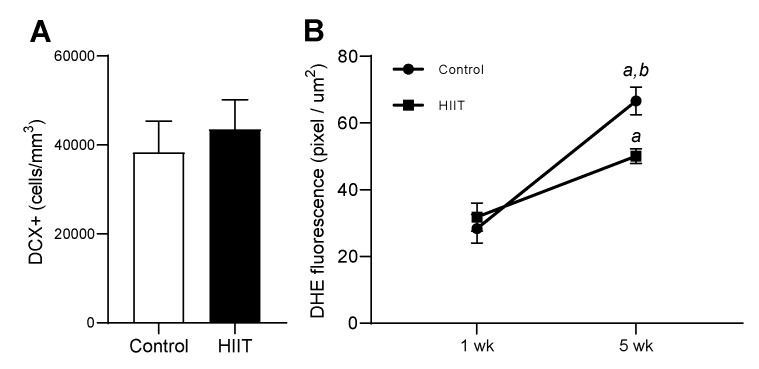
Effect of five-week HIIT on the DCX^+^ cells and SO levels in dentate gyrus. (**A**) Quantification of DCX^+^ cells and in the dentate gyrus (control *n* = 10; HIIT *n* = 10). (**B**) Superoxide anion levels in the dentate gyrus: 1 wk—HIIT for one week, 5 wk—HIIT for five weeks. Statistical analyses were performed by the Student’s *t*-test. * *p* < 0.05 (Figure A), and Analysis of variance (ANOVA) one-way Bonferroni post-hoc (Figure B), ^a^ *p* < 0.05 vs. Control 1 wk; ^b^ *p* < 0.05 vs. HIIT 1 wk.

In order to verify if the alterations observed at one-week HIIT remained a long time, markers of hippocampal plasticity (DCX) and redox status were investigated after five weeks of HIIT. As at one-week, five-week HIIT did not promote changes in the production of H_2_O_2_ in the hippocampus (Figure 5A) and cortex (Figure 5B), nor changes in a marker of oxidative stress (Figure 5C). However, especially in the dentate gyrus, the SO level was significantly lower in the trained group (Figure 5D,E) and the DCX^+^ cell content presented no significant differences between the control and trained animals (Figure 6A). Interestingly, comparing the SO levels in the dentate gyrus with time (from one to five weeks), a significant increase in SO in the non-trained condition was observed, an alteration probably related to aging [45], which was prevented in the HIIT trained animals (Figure 6B).

## 4. Discussion

Here, we investigated the exercise-related neuroplasticity in a model of high intensity interval training (HIIT) based on redox mechanism. We demonstrated that one week of HIIT modulated neuroplasticity increased mitochondrial content, unchanging levels of ROS production, or oxidative stress markers in the hippocampus of mice.

Mass spectrometry analysis, a powerful tool in the field of structural biology, showed that one week of HIIT significantly increased the biomarkers for cell proliferation and differentiation in the hippocampus. The MCM2 protein, increased after training, is a key protein in the cell replication complex, controlling DNA replication [42]. As MCM2 expression starts in the early G1 phase and is maintained throughout the cell cycle, this protein is considered a useful marker for detecting slowly cycling putative neural stem cells in situ [46]. MCM2 is present in higher levels than the short-lived proliferation marker Ki-67 [47], which may be the reason we did not detect changes in Ki-67 content after one-week of HIIT. In addition, HIIT increased DCX content, a marker of cells between a late progenitor cell stage and the early postmitotic stage (immature neurons), which correlates well with net neurogenesis [48,49,50]. DCX-expressing cells have been found upregulated after 10 days of voluntary wheel running in mice correlating with markers of a transient postmitotic stage of granule cell development in the adult hippocampus [50]. Another important neuroplasticity factor increased by HIIT was BDNF, which has been demonstrated to promote new glial [51], neuronal [52], and synaptic [53] formations. Many studies have shown that long-term interventions with exercise lead to an increase in BDNF, making this neurotrophin an important factor in exercise-induced neuroplasticity [4,54]. Corroborating this hypothesis, western blotting analyses showed increased Akt phosphorylation, which contributes to mediate cellular processes including cell proliferation and growth [55]. In brain, the Akt pathway mediates the effects of exercise, since the inhibition of the PI3K-Akt signaling pathway prevents exercise-induced synaptic plasticity and neurogenesis in the dentate gyrus [56]. Moreover, Akt signaling deregulation in the brain is linked with neuronal diseases [55,57].

In addition, one-week HIIT increased mitochondrial content in the hippocampus, verified by a high content of VDAC, an exclusively mitochondrial protein. Neuronal differentiation of postmitotic neurons from neural progenitors is frequently associated with an increase in mitochondrial mass per cell [58,59], respiratory capacity, and mtDNA upregulation [60]. In this step, neurite outgrowth, to become axons or dendrites, is an essential process in the formation of functional neuronal circuits (for a review, see [10]) and requires an active mitochondria (in number, function, distribution and shape) to energy supply (ATP and NADH) to enter into growing formed-axons [61,62] and to regulate subcellular Ca^2+^ homeostasis [62]. Chemical inhibition of mitochondrial translation by chloramphenicol [58] or accumulation of mtDNA damage [60] may prevent neuronal differentiation as well as mitochondrial dysfunction in aging that contributes to impaired neurogenesis [12,13]. In agreement, in continuous voluntary exercise, which is considered the strongest pro-neural trigger of hippocampal neurogenesis [63,64], an acute increase in mitochondrial content was also found, especially in dendritic segments, in addition to the increased presence of globular mitochondria and enhanced distribution of mitochondria to the dendritic arbor [65].

Finally, one week of HIIT did not promote changes in the redox state or excessive oxidative damage, verified by redox markers H_2_O_2_ and PCO in the hippocampus or SO levels specifically in the dentate gyrus. Clearly, between ROS generation and irreversive protein carbonilation, there is a series of cysteine residues that undergo reversible oxidation–reduction such as thiols (protein thiols, glutathione, and associated disulfides), where glutathione is the most important modulator of redox processes. However, different pools of glutathione are present in intracellular compartments, playing multiple functions and suffering complex regulation of its biosynthesis, utilization, degradation, and transport (for review, see [66]). Despite the known role of oxidative stress in a broad variety of central nervous system diseases, the role of ROS in neurogenesis is not completely understood, much less the effects of exercise in neural redox balance. Reports have shown that proliferative neural stem cells produce high endogenous ROS levels that regulate self-renewal and neurogenesis in a PI3K/Akt-dependent manner [67]. However, others have suggested that in these cells, the oxidized state is generated during a transient window of elevated neurogenesis accompanying normal neurogenesis, a stage characterized by an immediate increase in mitochondrial content and overall ROS production [68]. In this regard, mitochondria is considered a key player in neuronal differentiation by regulating the redox balance [13]. Evidence indicates that mitochondria may generate rapid bursts of SO (SO flashes), mediated by mitochondrial permeability transition pore (mPTP) opening, as a second messenger for neuronal differentiation [67,68,69]. In this sense, the decreased content of SOD2 antioxidant enzyme found in the hippocampus after one-week HIIT is an adaptation that could favor ROS burst; however, we cannot state this. Nevertheless, ROS production has to be accurately regulated since ROS overload may prevent intermediate progenitor cells differentiating into neuroblasts [13,69] and impair adult neurogenesis during aging [70]. Physical exercise, parallel to neurogenesis, has been shown to reduce oxidative stress in the hippocampus, as found after two weeks [71], 3–4 weeks [20,72], 6–8 weeks [73,74], or up to three months [16] of moderate aerobic exercise. Nonetheless, investigations into HIIT linked neurogenesis are still scarce and, heterogeneous experimental designs have begun to challenge the understanding of the HIIT-effect on neuronal function. In studies that analyzed oxidative stress, it was observed that, in the hippocampus, HIIT for six weeks reduced hippocampal oxidative stress by decreasing lipoperoxidation and inflammatory markers as well enhancing antioxidant defenses after six weeks of training [27]. In corroboration, a single HIIT session also demonstrated the ability to increase antioxidant defense, measured from the activity of SOD in the hippocampus [75]. Additionally, while no changes in oxidative stress were observed in the cortex after six weeks of high-intensity interval training, in the cerebellum, there was an increase in both SOD activity and lipid peroxidation [26]. Conversely, six weeks of HIIT increased oxidative stress in the brain, despite the increase in neurotrophins [76]. Additionally, in experimental designs that evaluated neuroplasticity, Nokia et al. demonstrated only a modest increase in immature neurons after seven weeks of HIIT [6]. Interestingly, HIIT associated with experimental ischemic stroke models was able to increase neuroplasticity (BDNF) [77,78] and enhance markers against depression [77]. In this regard, our experimental design evaluated both oxidative stress and neuroplasticity, and the HIIT protocol presented a positive effect when maintained long-term (one to five weeks), thus mitigating SO levels in the dentate gyrus and preventing this elevation over time. Although increased expression of DCX has been observed in the hippocampus, the level of DCX^+^ cells in the dentate gyrus was similar to that found in non-trained animals. These results suggest that in the long-term, the antioxidant effect of HIIT did not influence the immature neuron markers. 

Although high-intensity interval training is an actual training-trend around the world, its impact on human neuronal function is poorly understood. In light of this, three months of HIIT increased glucose uptake by the brain of both young adults and the elderly [79]. However, HIIT demonstrated a reduction in opioid receptors in brain regions linked to mood and correlated with a reduction in the feeling of satisfaction after the exercise session [80]. There was also an increase in brain damage markers after HIIT sessions [81]. Thus, the diversity of experimental designs on the effects of HIIT on brain functions makes the results controversial and more investigations are needed for a better understanding of the HIIT and their effects in neuronal function.

## 5. Conclusions

Our data showed that HIIT for one week could increase neuroplasticity associated with increments in mitochondrial content, regardless of changes in redox status. Our results shed light on the neuroprotective effects of HIIT training as well as insights for future investigations linked to metabolic changes in the differentiation and maturation process of neuronal cells.

## Supplementary Materials

The following are available online at https://www.mdpi.com/2076-3921/9/5/445/s1, Table S1: List of proteins analyzed by LC-MS/MS.
