# Supplementary material for: One-Week High-Intensity Interval Training Increases Hippocampal Plasticity and Mitochondrial Content without Changes in Redox State
Source: Antioxidants (Basel). 2020 May 21;9(5):445. doi: 10.3390/antiox9050445 (PMC7278594; doi:10.3390/antiox9050445)
Supplement: Supplementary file 1 [file antioxidants-09-00445-s001.pdf]

Supplementary Table S1. List of proteins analyzed by LC-MS/MS

Protein	Precursor peptide (Seq.)	z	*m/z*	R. T.	L. P.	N. F.	Fragmented Ions (Sequence, z, *m/z*)
SOD2	GDVTTQVALQPALK	2	721332431	9,89	75-88	3	QVALQPALK, 1,967593492 – VALQPALK, 1, 839534915 – VALQPALK, 1, 740466501
KI67	ESGELSEGSEK	2	576578481	9,44	869-879	3	GELSEGSEK, 1, 935431632 - LSEGSEK, 1, 749367575 - SEGSEK, 1, 636283511
Hur	DANLYISGLPR	2	610190766	13,04	104-114	3	LYISGLPR, 1, 918540728 - YISGLPR, 1, 805456664 - YISGLPR, 1, 642393336
Syn1	GSHSQSSSPGALTLGR	3	514884429	4,82	430-445	3	PGALTLGR, 1, 784467563 - GALTLGR, 1, 6874148 - LTLGR, 1, 559356222
Glast	TTTNVLGDSLGAGIVEHLSR	3	681089923	17,4	479-498	3	GAGIVEHLSR, 1, 1038569068 - GIVEHLSR, 1, 910510491 - VEHLSR, 1, 740404963
MAP2	LINQPLPDLK	2	576195121	11,21	1662-1671	3	NQPLPDLK, 1, 924514908 - QPLPDLK, 1, 81047198 - PLPDLK, 1, 682413403
NeuN	GFGFVTFENSADADR	2	817354996	16,1	155-159	2	TFENSADADR, 1, 1125480707 - FENSADADR, 1, 1024433028 - ENSADADR, 1, 877364615
Nestin	ADDELAALR	2	487529636	8,25	55-63	3	DDELAALR, 1, 902457787 - ELAALR, 1, 672403901 - LAALR, 1, 543361307
DCX	GNPSAAAGPK	2	435477076	5,6	275-284	3	NPSAAAGPK, 1, 812426093 – SAAAGPK, 1, 601330401 – AAGPK, 1, 443261259
BDNF	DADLYTSR	2	470993071	12,79	87-94	3	DLYTSR, 1, 754372994 - LYTSR, 1, 639346051 - YTSR, 1, 526261987 -
MCM2	GLALALFGGEPK	2	587198031	14,62	493-504	3	LALFGGEPK, 1, 931524744 - LFGGEPK, 1, 747403566 - FGGEPK, 1, 634319502
VDAC	LTFDSSFSPNTGK	2	701256486	10,92	109-121	3	DSSFSPNTGK, 1, 103946908 - SSFSPNTGK, 1, 924442137 - FSPNTGK, 1, 75037808 -
α-tubulin	AVFVDLEPTVIDEVR	2	851970081	19,13	64-78	3	EPTVIDEVR, 1, 1057552415 – PTVIDEVR, 1, 928509822 – VIDEVR, 1, 73040938

Note: R.T. = Retention time (minutes); L. P. = Location on protein; N. F. = Number of fragments.
